# Atopic Dermatitis Studies through *In Vitro* Models

**DOI:** 10.3389/fmed.2017.00119

**Published:** 2017-07-24

**Authors:** Evelyne De Vuyst, Michel Salmon, Céline Evrard, Catherine Lambert de Rouvroit, Yves Poumay

**Affiliations:** ^1^Cell and Tissue Laboratory, URPhyM-NARILIS, University of Namur, Namur, Belgium; ^2^StratiCell, Les Isnes, Belgium

**Keywords:** atopic dermatitis, *in vitro*, model, reconstructed epidermis, skin equivalents, epidermal keratinocytes

## Abstract

Atopic dermatitis (AD) is a complex inflammatory skin condition that is not fully understood. Epidermal barrier defects and Th2 immune response dysregulations are thought to play crucial roles in the pathogenesis of the disease. A vicious circle takes place between these alterations, and it can further be complicated by additional genetic and environmental factors. Studies investigating in more depth the etiology of the disease are thus needed in order to develop functional treatments. In recent years, there have been significant advances regarding *in vitro* models reproducing important features of AD. However, since a lot of models have been developed, finding the appropriate experimental setting can be difficult. Therefore, herein, we review the different types of *in vitro* models mimicking features of AD. The simplest models are two-dimensional culture systems composed of immune cells or keratinocytes, whereas three-dimensional skin or epidermal equivalents reconstitute more complex stratified tissues exhibiting barrier properties. In those models, hallmarks of AD are obtained, either by challenging tissues with interleukin cocktails overexpressed in AD epidermis or by silencing expression of pivotal genes encoding epidermal barrier proteins. Tissue equivalents cocultured with lymphocytes or containing AD patient cells are also described. Furthermore, each model is placed in its study context with a brief summary of the main results obtained. In conclusion, the described *in vitro* models are useful tools to better understand AD pathogenesis, but also to screen new compounds in the field of AD, which probably will open the way to new preventive or therapeutic strategies.

## Introduction

Atopic dermatitis (AD), also referred to as atopic eczema, is one of the most common chronic inflammatory skin diseases and the most common form of eczema in childhood. AD is characterized by dry erythematous lesions and intense pruritus. At the histological level, acute AD lesions mainly involve the epidermis and typically exhibit spongiosis in the suprabasal epidermal layers when compared to the normal tissue. Spongiosis is due to alteration in cohesion between keratinocytes, resulting in enlarged intercellular spaces suggestive of intercellular edema. Inflammatory infiltrate (predominantly lymphocytes) can also be noticed at the epidermal level. Lesional dermis is characterized by a marked perivascular T cell infiltrate, predominantly composed of activated memory/effector T cells. Eosinophils and mast cells (presenting variable degranulation stages) can be observed while basophils and neutrophils remain scarce. In chronic stages of AD, lichenified lesions appear, typically exhibiting epidermal hyperplasia and parakeratotic hyperkeratosis which correspond together to thickening of spinous and cornified layers. A high number of immunoglobulin E (IgE)-bearing Langerhans cells are present inside the epidermis. Slight infiltration by lymphocytes and macrophages, together with elevated number of mast cells, generally fully granulated, characterize the inflammatory dermal infiltrate. More numerous eosinophils can also be observed inside the dermis of chronic AD skin lesions (1, 2).

Regarding its pathogenesis, AD is a relapsing disease that is complex, multifactorial, and still not completely understood. AD can very likely be triggered by both epidermal barrier alterations and Th2 immune response dysregulation, each of them being potentially responsible for the induction of the other alteration, thereby creating a vicious circle responsible for the lesions (3–5). Understanding the pathogenesis of AD is further complicated by several genetic factors, as well as by diverse environmental factors like intensive use of soaps and detergents, house dust mites, pollutants, allergens, and psychological stress (6–8). Regarding genetic predisposition, filaggrin (FLG) gene has been extensively studied for variations linked to the context of AD (9–14). Indeed, FLG is thought to play a crucial role in the pathophysiology of AD because the protein encoded is involved at different levels in the formation and maintenance of a correct epidermal barrier (15, 16). Indeed, any decrease in FLG expression, as well as in its function, may alter aggregation of keratin filaments and thus formation of functional corneocytes. *Via* its metabolites, FLG alterations may affect the levels of natural moisturizing factors (NMF) and consequently modify skin hydration and possibly its pH values. Dehydration of the skin induces xerosis, leading then to pruritus and to further alterations of the epidermal barrier, whereas any elevation in the skin surface pH enhances activity of proteases responsible for the desquamation process and decreases activity of enzymes implicated in barrier lipid synthesis (17). All these altered processes can weaken the epidermal barrier and enhance penetration of allergens/pathogens, inducing further skin inflammation. It is important to note that FLG loss-of-function mutations are not the only reasons responsible for alterations in the role of FLG in epidermal barrier formation and maintenance. Indeed, AD patients present alteration of their epidermal barrier irrespectively of their FLG genotype (18). Further, a relationship between FLG and AD has been proven independent from FLG mutations within a French cohort (19) for instance, whereas strong reductions in FLG expression levels are observed in AD skin (lesional and non-lesional) (20, 21). Pellerin and co-workers reported that *in vitro* treatments of keratinocytes with inflammatory cytokines highly expressed in AD epidermis, namely IL-4, IL-13, and IL-25, were found to reduce FLG expression. Similar findings have been reported by further groups (20, 22, 23) and other cytokines like IL-17, IL-22, or IL-31 also were found potentially able to decrease FLG expression in keratinocytes (24–26). Thus, decrease in FLG contribution to the epidermal barrier, either through reduced expression or by loss-of-function gene mutations, are strongly related to inflammatory conditions. Any downregulation of FLG in the epidermis worsens barrier permeability and consequently results into triggered inflammation, particularly in lesional areas.

Thus, this limited, but quite well-studied, part of AD pathogenesis provides very useful information to better understand initiation and maintenance of the afore-mentioned vicious circle and likely the particularly relapsing nature of the disease.

**Figure 1:**
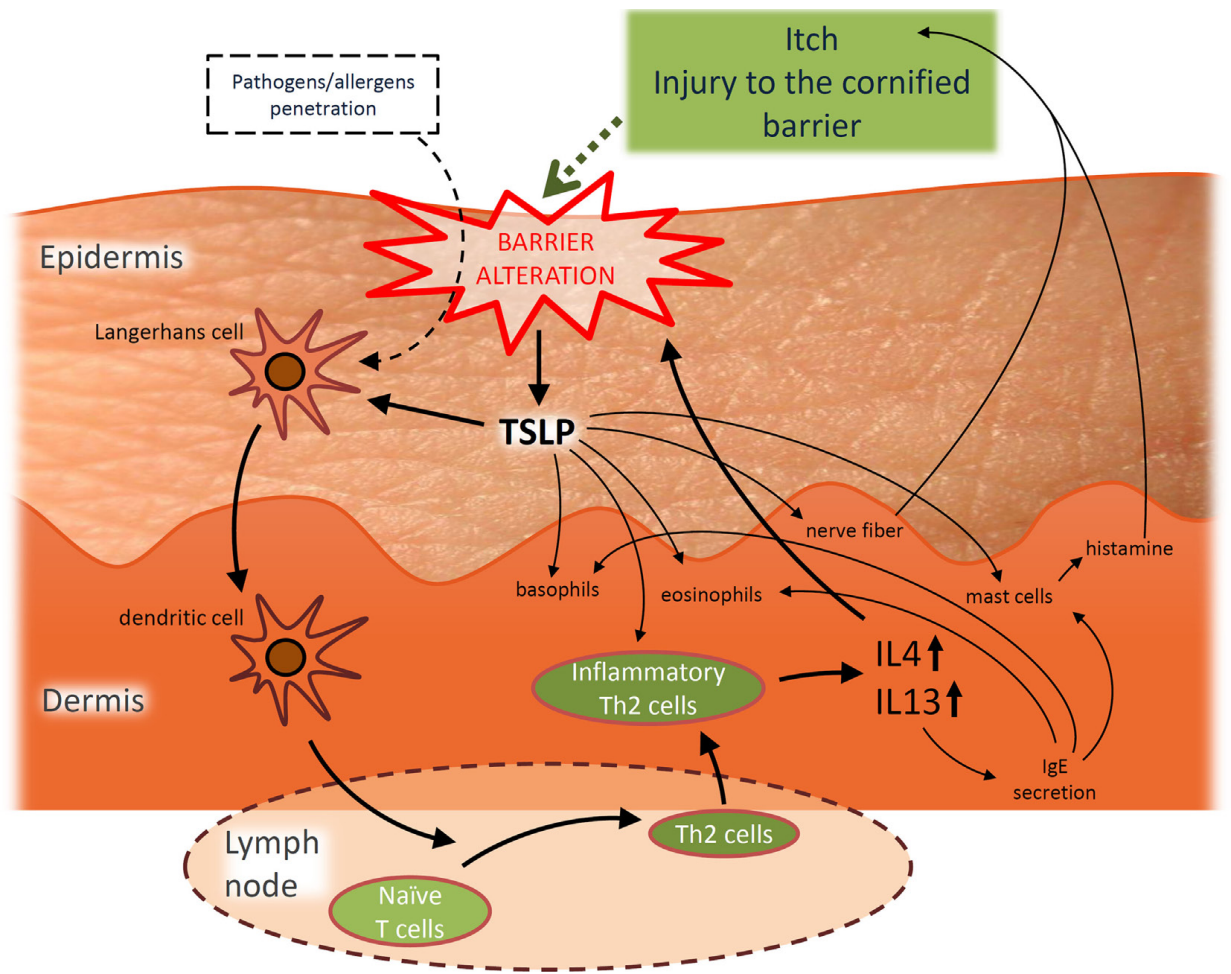
Hypothetic simplified representation of the pivotal role played by thymic stromal lymphopoietin (TSLP) in the pathogenesis of acute atopic dermatitis (AD) and the interplay between barrier alterations and Th2 immune dysregulation. TSLP released from challenged keratinocytes is able to activate migration of Langerhans cells toward draining lymph nodes (35) where they subsequently initiate allergic response by promoting differentiation of naïve CD4^+^ T cells into inflammatory Th2 effectors. TSLP can also directly act on these Th2 effectors to induce their proliferation (43–45). Th2 effector cells produce IL-4, IL-5, IL-13, TNFα, while downregulating IFNγ (35), possibly explaining the link observed between barrier alterations and inflammatory conditions. Indeed, among these cytokines, IL-4 and IL-13 are more particularly known as being able to alter the expression of proteins involved in the epidermal barrier like filaggrin (19, 20, 49), loricrin (LOR) and involucrin (IVL) (50), desmoglein 1 and 3 (component of desmosomes) (49, 51), sphingomyelinase and glucocerebrosidase (genes implicated in the synthesis of ceramides) (52), or the tight junction component claudin-1 (53). Every cytokine-induced alteration of the epidermal barrier allows conditions more favorable for penetration of allergens/pathogens that can then be recognized by dendritic antigen-presenting cells. Processed allergens/pathogens are next presented to naive B cells and activate them to eventually produce specific immunoglobulin E (IgE). Such IgE then bind to the high affinity receptor FcεRI expressed on basophils, eosinophils, and mast cells. After re-exposure to a previously encountered antigen, the body can respond directly and more strongly. Indeed, when IgE are produced in greater amounts, basophils, eosinophils, and mast cells are ready to mediate inflammatory reactions. This immune inflammatory response is known as the allergic response. Basophils are able to promote Th2 cytokine response, while mast cells can massively degranulate, notably releasing histamine which induces edema and pruritus. IL-4 is known to modulate this allergic response and to induce overproduction of IgE. In addition, TSLP could also play a role in the itch symptoms observed in AD patients. Indeed, TSLP activates mast cells to release histamine upon binding to its specific receptor. Even further, keratinocytes directly interact with dermal sensory neurons *via* TSLP, thereby inducing itch (42). Moreover, TSLP may also promote basophil and eosinophil responses. Basophils promote Th2 cytokine response, while TSLP induces recruitment of eosinophils to Th2 cytokine-associated inflammation sites (54).

In addition, AD is considered the initial step of the so-called “atopic march,” which corresponds to the consecutive development of asthma and/or allergic rhinitis during the life-course of 30–50% of AD patients, particularly those who are the most severely affected (27–29). The “atopic march” can partly be explained through the role played by thymic stromal lymphopoietin (TSLP), a cytokine induced by trauma, microbial infection, toll-like receptor activation, or by combinations of inflammatory cytokines (30–34). TSLP is found at elevated concentrations in AD epidermis (35) and its induction in skin has been reported to be accompanied by elevated levels in blood circulation. TSLP is believed to be a systemic driver for bronchial hyperresponsiveness since blockade of TSLP signaling (36) or inducible gene deletion of TSLP in mice keratinocytes (37, 38) prevents occurrence of the atopic march, suggesting a potential direct link between AD and allergic asthma and/or allergic rhinitis (39–41). Of further interest, TSLP released from challenged keratinocytes (31–35) would play an important role in the disease by contributing to the itch symptoms that characterize the disease (42) but also to the Th2-promoting conditions (43–47), thus favoring inflammation and thereby also barrier alterations (48), playing thereby a potentially initiating role in the vicious circle of AD pathogenesis. The particular role played by TSLP is described in Figure 1.

Atopic dermatitis is mostly known as an imbalance of the Th2 immune response as in the acute phase of the disease significant increases in gene expression levels of major Th2 cytokines are reported. The disease itself is rather characterized by a biphasic inflammation and a switch to a Th1 environment is commonly reported during chronic phases of AD (1, 55–57). However, situation is probably more complicated as small increases in Th1 and Th17 are also found in acute lesions while a progressive activation of Th2 and Th22 cells would also characterize the chronic phase of the disease (58).

In regard to the diversity of factors involved in AD pathogenesis, it is currently difficult to elaborate treatments that can directly target all aspects involved in the etiology of the disease. Existing treatments are aimed at restoring epidermal barrier defects and/or reducing skin inflammation, in combination with avoidance of environmental factors that are supposed or demonstrated to negatively influence the severity of the disease (59). Studies that more deeply investigate mechanisms leading to this pathology are, therefore, still required. In order to further investigate the development of AD lesions, as well as for preclinical development of potentially protecting or resolving treatments and drugs, animal models that adequately reproduce symptoms of AD have been first generated [for a review see Ref. (60)]. However, mice do not spontaneously develop AD and translation of the disease condition from experimental mice to humans and *vice versa* remains questionable because, even though mouse and human skin exhibit numerous similarities, crucial differences do also exist. For instance, mouse keratinocytes produce fewer epidermal layers (6–7 layers for mouse versus 16–18 layers for human) and renewal of the murine tissue happens within 8–10 days only, whereas the one in humans lasts for 28 days or so. Obviously, mouse skin contains higher numbers and wider diversity of hair follicles (less present in tail and ears), and epidermal papillae (also known as *rete ridges*) penetrate less deeply. Mouse skin also lacks sweat glands (eccrine sweat glands are exclusively present in the pads) and melanocytes in the interfollicular epidermis. Regarding potential explanation of mechanisms driving inflammatory skin pathologies, it is also important to take into account the differences in the immune cell population. Indeed, γδ T cells as well as dendritic epidermal T cells (DETC) are present in mouse but not in human epidermis. These DETC can secrete large amounts of pro-inflammatory mediators involved in epidermal communication with keratinocytes and Langerhans cells (61, 62).

In conclusion, *in vitro* models based on human tissues to study etiological parameters of inflammatory cutaneous diseases like AD have been awaited for a long time, but such models are now regularly reported in several various context. In addition for this requirement of human-based *in vitro* models for etiological studies, it worth mentioning that, since authorities of the European Union have prohibited the testing of cosmetic finished products or of their ingredients on laboratory animals and they even banned the marketing of cosmetic products tested on animals outside the EU (Regulation (EC) No 1223/2009), legal restrictions have further argued for the development and assessment of *in vitro* models that involve cutaneous tissues. Nowadays, *in vitro* models have to become reliable alternative methods for studies about human epidermal pathologies, as well as for screening eventual toxic properties on skin of still unevaluated or new chemicals.

Using *in vitro* model, it is possible to mimic different characteristics of the AD pathology. Indeed, histological features of the AD epidermis can be induced, alteration of the epidermal barrier can be monitored, some characteristics of inflammation can be reproduced and expression of genes can be easily analyzed both at the mRNA and protein levels. Several different *in vitro* models regarding AD have been published within the last 10 years in the literature. Given the complex interplay between immune dysregulations and epidermal barrier alterations in AD, most *in vitro* models have to focus on these two aspects. However, limitations emerge with each model when scientists are looking for the best reproduction of the pathology in order to analyze very specific aspects or to screen new drugs/cosmetics for potential propensities to improve critical aspects of the diseased skin. This review first aims at summarizing features of AD that can be satisfactorily mimicked *in vitro* and then provides an update about several published *in vitro* models able to bring some useful information about AD pathology. It is, however, important to note that this review does not pretend itself to be exhaustive about published *in vitro* models of AD. It rather focuses on models developed to study epidermal characteristics of the disease. For each mentioned model, important results reported after the use of these models are described but information remains limited to key observations.

## *In Vitro* Models of AD

### Two-Dimensional Culture Systems

Cultures of a single cell type, either immune cells or keratinocytes, may be consider as the simplest *in vitro* models available for analysis of cellular phenomena involved etiological processes of epidermal inflammatory diseases. Of course, such models do not, however, take into account complex structures and interactions within tissues, but present the enormous advantage to allow precise evaluation of a specific cell response toward any given challenge.

### Immune Cells

In order to understand parameters regulating roles and abundance of lymphocytes, mast cells, eosinophils, and other immune cell types in AD-patients skin, it can be of interest to study each cell type, one after the other, to characterize their reaction to different stimuli and environmental conditions. Immune cells implicated in the pathogenesis of AD can be isolated from peripheral blood of AD patients or normal subjects. For instance, purified lymphocytes can be studied *in vitro*, allowing thereby to analyze gene expression of surface receptors or investigating cell responses toward specialized cytokines or any other potentially regulating ligand. For example, it was observed using this type of investigation that the frequency of lymphocytes expressing the TSLP receptor was correlated with severity of AD disease (63).

Mast cells can also be investigated *in vitro* after isolation and culture in serum-free methylcellulose media containing stem cell factor, IL-6, and IL-3. Initially, this type of study has been conducted in order to compare proliferation of mast cells isolated from donors with normal IgE serum levels, to those isolated from AD patients with high serum IgE levels. Both mast cell populations exhibited no highlighted difference in proliferation and similarly released histamine. Furthermore, significantly enhanced histamine release was observed for both types of mast cell donors treated with IL-4, suggesting that regulation of mast cell function rather happens through environmental stimulation by interleukins, than by genetic predisposition in the case of AD (64). Conversely, while the number of colonies made of mast cells was unchanged, eosinophils and basophils from AD donors did exhibit enhanced growth potentials (64).

Basophils or eosinophils, both recognized as actual effectors for allergic inflammation, have also been analyzed through *in vitro* techniques by other investigators. For instance, fibroblasts were investigated as monolayers in the presence of culture medium containing basophils or eosinophils. Such studies interestingly highlighted the requirement for direct intercellular contacts during interactions between basophils and fibroblasts, while soluble mediators were found able to mediate cross talk between eosinophils and fibroblasts. These types of interaction in presence of toll-like receptor 2 ligands (e.g., *Staphylococcus aureus*) could thus be considered responsible for secretion of pro-inflammatory cytokines, possibly participating thereby in inflammatory responses that are typically exacerbated during *Staphylococcus aureus* infections (65).

### Keratinocyte Monolayer Cultures

Using culture of cell monolayers, some epidermal differentiation of keratinocytes can be achieved by either culture confluence (66) or increasing the calcium medium concentration (67), or by both. Such cultures are easily performed and remain very useful for studying simple basic molecular mechanisms. Incubation of either HaCaT cells (49) with IL-4 and IL-13 (10 ng/ml each cytokine) or primary human keratinocytes (20) with IL-4 and IL-13 (50 ng/ml each), two Th2 cytokines overexpressed in AD skin, have been performed to identify their effects on such cells. Indeed, during incubation with these cytokines, epidermal cells exhibit a notably reduced gene expression for FLG. Further, Omori-Miyake and colleagues demonstrated a downregulation of expression of keratin 1 and 10, desmoglein 1 and desmocollin-1 *via* STAT6-dependent mechanisms transduced *via* IL4RA. In addition, this type of analysis allows the demonstration for instance that silencing FLG expression using shRNA interference can simultaneously induce alterations in the amounts of synthesized cornified envelop-related proteins [expression of keratin 5, 10, and 14, IVL, and transglutaminase-1 is decreased, while the one of loricrin (LOR) is upregulated]. Interestingly, FLG silencing does also result in increased release of Th2 cytokines IL-4, IL-5, and IL-13, while production of IFNγ is decreased (68).

Mainly used to answer mostly basic questions regarding the biology of particular cell types, cultures of monolayers are broadly investigated because they are easy to perform, allow simple analytical probing, and are thus efficient at providing results with potential significance. Such cultures of human keratinocytes have indeed brought methods to test new ideas, as well as data suggestive for explanation of still obscure phenomena. For instance, while trying to understand in monolayer cultures of keratinocytes how cell signaling produced by cholesterol depletion was affecting their phenotypes, transcriptomic homologies were found between such keratinocytes and keratinocytes analyzed inside AD lesions (69). In other words, a study dealing with plasma membrane lipid microdomains opened up questions about their potential relevance and/or relevance of consequences linked to their signaling properties in order to understand and investigate AD.

Still, epidermal keratinocytes isolated from AD patients can also be cultured and compared to cells of healthy donors. In such cultures of keratinocytes, the profile for chemokine production inside cells from non-lesional areas of AD patients can be compared to normal keratinocytes (70, 71). It was reported this way that keratinocytes from AD patients spontaneously express elevated levels of granulocyte macrophage colony-stimulating factor (71). Keratinocytes from AD patients can further be used to evaluate how cytokines affect their phenotype. For example, IL-4, IFN-γ, and TNF-α were reported to trigger the production of other chemokines, like IL-8, C–C motif chemokine ligand 5, RANTES, or C–X–C motif chemokine ligand 10 in keratinocytes from non-lesional AD skin (70).

Although keratinocytes cultured as immerged monolayers are simple and useful research materials, they nonetheless do not stratify and neither produce an efficient barrier. Therefore, the development of epidermal *in vitro* models that produce a functional barrier has become crucial to screen treatments affecting this barrier in preclinical studies.

## Three-Dimensional Skin Models

In order to achieve the production of an efficient epidermal barrier, keratinocytes have to be exposed at the air–liquid interface while being cultured in conditions that favor their stratification. Two models are nowadays commonly available for studies of epidermal properties, reconstructed human epidermis (RHE) and full-thickness human skin equivalents (HSE), both closely mimicking the *in vivo* characteristics of the human epidermis in terms of differentiation, barrier function, and histology (72–77). RHE are composed of keratinocytes only, grown at the air–liquid interface on inert polycarbonate filters. Conversely, HSE are based on dermal-like matrix initially organized before seeding growing keratinocytes on top for epidermal stratification and keratinization by exposure to air. The dermal-like matrix can either be based on de-epidermized dermis or on collagen matrices containing fibroblasts. Three different approaches can be used for this last purpose (78):

(1) The gel approach consists in seeding fibroblasts in extracellular matrix (ECM) components such as collagen, the major element of the dermis.
(2) Dermal substitutes can also be obtained by culturing fibroblasts on scaffolds made of synthetic or biological materials (79). Collagen mimics many of the structural properties of the ECM and can be used as biological scaffold in which fibroblasts are then cultured. However, manufacturing a collagen scaffold having a precisely controlled 3D pore structure, which can be important for cell proliferation and migration, is not an easy task. Mechanical properties of scaffolds can be improved by using a cryogenic dispenser system (80) together with the use of cross-linking chemical agents like glutaraldehyde (81) or biological agents like chitosan that are then mixed with collagen (79).
(3) Another possibility is to prepare dermal substitutes thanks to the capacity of fibroblasts to secrete their own ECM *in vitro*. After about 28 days of culture, fibroblasts are embedded into their own ECM and cell sheets, that can be manipulated, are obtained (79).

Both, RHE and HSE models, present advantages and disadvantages. Obviously, HSE mimics the epidermis as well as the dermal compartment. Further, as diffusion of lipophilic substances (e.g., lipid-based creams) is more efficient *in vivo* than *in vitro* in skin equivalents, adding a dermal-like compartment allows partial improvement *in vitro* (82). However, their production takes longer and is more complex. On the other hand, main advantages of RHE arise from the fact that they allow evaluation of keratinocyte-specific cell type response. In particular, amounts of molecules released or secreted by keratinocytes in culture medium can be measured using ELISA-like techniques.

Interestingly, *in vitro* models can be altered in ways that mimic AD conditions at the epidermal level, either by incubation with cocktails of interleukins overexpressed in the pathology or by silencing gene expression of components involved in development and structure of the barrier. Finally, keratinocytes, as well as other cells types isolated from AD patients, can also be used in models.

### 3-D Challenged Models

Several different cytokines sets have been chosen to create barrier alterations inside cutaneous *in vitro* models. Depending on the cytokines, cell regulations are activated in keratinocytes and demonstrate that this kind of treatments can be able to induce phenotypic features that recall epidermal lesions seen in AD lesions, like widening of intercellular spaces (spongiosis), alteration of expression and localization of differentiation markers, and/or modified lipid organization.

For instance, HSE generated on de-epidermized dermis were stimulated with IL-4 and IL-13 from day 10 until day 13 of reconstruction of the epidermis, using a concentration of 30 ng/ml for each interleukin (83). IL-4 and IL-13 are two Th2-type cytokines that exhibit elevated expression in AD lesional skin (20, 23). The treatment induced spongiosis, apoptosis, and increased expression of genes that become specifically expressed in AD epidermis, like carbonic anhydrase II (CA2) and neuron-specific Nel-like protein 2 (NELL2) (10, 84). Conversely, the treatment was unable to trigger the expression of psoriasis-associated genes, like human beta defensin 2 (hBD2) and elafin. Several other studies as well have investigated the epidermal consequences produced by incubation with these two Th2 interleukins, eventually in combination with other inflammatory molecules. For example, IL-25 was added to IL-4 and IL-13 because this cytokine can act on both immune cells (85) and keratinocytes, where it can reduce expression of FLG (19), explaining thereby the systematic link observed between inflammation and barrier disruption in AD lesions (22, 86). Consequently, IL-4 and IL-13 (50 ng/ml each) were also combined with IL-25 (20 ng/ml) in a study performed on RHE model during 48 h (87). Interestingly, the epidermal consequences produced by these cytokines were enhanced when cholesterol was depleted from the plasma membrane of keratinocytes ahead of the interleukin treatment, suggesting that plasma membrane lipid microdomains disruption would render keratinocytes more sensitive to the Th2 interleukins. In such model, spongiosis and hypogranulosis were observed together with alterations in the expression of specific AD-markers [FLG, LOR, CA2, NELL2, TSLP and hyaluronic acid synthase 3 (HAS3)], as well as in barrier functions, as highlighted by transepithelial electrical resistance measurements and lucifer yellow permeation tests (87). In other studies, Th2 cytokines (IL-4 or IL-13 at 100 ng/ml each) combined with pro-inflammatory cytokines like TNF-α (20 ng/ml) or IL-1α (100 ng/ml) for 48 h have been shown to act synergistically in order to induce production of TSLP in human skin explants (88). In a RHE model, IL-4, IL-13 (30 ng/ml each), and TNF-α (3.5 ng/ml) were further combined to IL-31 (15 ng/ml), a pruritus-related cytokine, and this cocktail induced AD-like features such as decreased expression of epidermal differentiation proteins like FLG and LOR, spongiosis, increased secretion of TSLP, and alterations of barrier properties that concern lipids (89). Indeed, lipid organization was affected as the treatment induced a decrease in the level of long chain free fatty acids and ester linked ω-hydroxy ceramides. Still in another study, IL-22 was combined to TNF-α, IL-4, and IL-13 and applied together as an “AD-mix” on RHE at day 10 of epidermis reconstruction for 48 h at a concentration of 3 ng/ml (90) in order to compare this set of interleukins with another “psoriasis-mix” set of interleukins containing IL-17, a psoriasis-related cytokine, instead of Th2 cytokines IL-4 and IL-13. Using the “AD-mix,” one can observe epidermal features of AD including decreased expression of FLG, small proline rich proteins (SPRR2A) and increased expression of IL-13RA2 (one of the IL-13 receptor subunit), together with a weak increase of S100 calcium-binding protein A7 expression. Whereas using the “psoriasis-mix,” lighter decrease in the expression of FLG is observed, together with a stronger increase of S100 calcium-binding protein A7 expression, and a weaker increase of SPRR2A and IL-13RA2 are reported.

Still another way to induce *in vitro* AD-like features in RHE was found while combining Poly I:C (10 µg/ml), a toll-like receptor 3 ligand mimicking viral double-stranded RNA, with TNF-α (10 ng/ml), IL-4, and IL-13 (50 ng/ml each) at day 14 of epidermis reconstruction and for 48 h (91). In this case, spongiosis, alterations of differentiation markers, increase in TSLP expression and IL-8 secretion was observed in the RHE, while its transcriptomic profile was reminiscent of the one observed in AD keratinocytes.

*In vivo*, AD epidermis is bathed by a complex mixture of cytokines that can produce overlapping, redundant, additive, or even opposite effects. Indeed, IL-4, IL-13, IL-22, and IL-25 are all able to downregulate expression of differentiation markers such as FLG (19, 22, 24, 86). Conversely, TNF-α exhibits only weak effects on the expression of keratinocyte differentiation markers. Nonetheless, TNF-α appears necessary, in combination with Th2 cytokines, in order to induce epidermal expression of TSLP. TSLP is released in response to a combination of different molecules like Th2 cytokines (IL-4 and -13) and pro-inflammatory cytokine (TNFα) or poly I:C combined to these cytokines. However, when present, IL-17 suppresses this upregulation (92). Regarding other opposite effects, both TNF-α and IL-22 induce expression of antimicrobial peptides such as hBD2, while Th2 cytokines IL-4 and IL-13 conversely decrease the levels of beta defensin 2 (93). Still more complicated, concentrations and timings of treatment with chosen cytokines seems to likely favor different types of tissue response. For now, the precise concentrations of these interleukins in skin of AD patients and in healthy skin are unknown yet. Thus, one must admit that currently available research data on *in vitro* models have been evaluated in regard of dose-dependent effects produced by those cytokines on the expression levels of proteins like FLG or TSLP inside the tissue.

Also interesting to mention, because pruritus is an important symptom of AD, other factors have been studied *in vitro* in order to better understand basic mechanisms regulating itch. An innervated skin model was set up for this purpose since increased innervation is another feature reported about AD skin (94, 95). Porcine dorsal root ganglia neurons were seeded in a collagen gel in top of which collagen gel comprising fibroblasts was added. Keratinocytes were then seeded on top of this scaffold in order to reconstruct an epidermis for 12 days at the air–liquid interface. That model has allowed to identify that neurons release a molecule called calcitonin gene-related peptide able to induce epidermal thickening (96). Besides increased innervation, elevated numbers of mast cells releasing histamine, among other molecules, are also part of AD skin. HSE cultured for 14 days were, therefore, incubated with histamine (10 µM) for the whole duration of reconstruction or only during the three first days. Decreased expression of differentiation markers such as keratin 1 and 10, FLG, LOR, tight junction proteins, and desmosomal proteins was found, likely responsible for alterations in the epidermal barrier function (97).

To summarize, IL-4 and IL-13 seem to be the most crucial, probably unavoidable, cytokines which can induce an AD-like phenotype in epidermis, *in vitro*. However, variable interleukin cocktails can be added, together with other inflammatory molecules or neuropeptides, depending on research interests and on which target genes treatments or analysis are focused.

### 3-D Knockdown Models

In both lesional and non-lesional skin of AD patients, FLG expression is decreased, due to loss-of-function mutations, or irrespective of any particular FLG genotype (19, 21). Organotypic skin models containing knockdown of FLG in keratinocytes have been set up. Two studies have analyzed FLG knockdown in HSE by means of siRNAs (98, 99). Kuchler et al. (87) showed that FLG silencing performed in keratinocytes used to produce HSE grown at the air–liquid interface for 14 days, disturbed the development of the stratum corneum and also induced spongiosis. An increased susceptibility to irritating exposure, as well as altered transepidermal absorption of testosterone, but not of caffeine, was also reported. Using siRNA as well but in HSE analyzed after 7 days of epidermis reconstitution, Mildner et al. (99) have described increased permeability of the epidermal barrier to the lucifer yellow dye, hypogranulosis, enhanced activation of caspase-3 after UVB irradiation, but no alteration of lipid composition or of keratinocyte differentiation in the knockdown tissue. While using an shRNA procedure to also suppress FLG expression in a RHE model grown for 11 days, Pendaries and collaborators (100) reported hypogranulosis and increased barrier permeability of the lucifer yellow dye, together with a reduced number of keratinocyte layers, reduced cornified layer thickness, and decreased levels of the NMF, potentially responsible for the increased UV sensitivity already reported by Kuchler and colleagues (98). However, unlike Mildner and co-workers (99), alterations were reported in the mRNA and protein expression of components deeply involved in the epidermal differentiation process (100). Indeed, filaggrin-2 (FLG2), LOR, caspase 14 and bleomycin hydrolase were altogether reported as reduced conversely to the increased expression of corneodesmosin (100). Knockdown of FLG using shRNA was also performed in HSE generated using N/TERT keratinocytes (101). In this model, no alteration is reported neither regarding epidermal morphology (keratin 10, LOR, and proliferation marker ki67 were analyzed), neither lipid composition nor epidermal permeability (butyl-PABA permeation studies were performed). The discrepancies reported by the different groups could probably be attributed to the fact that different targets/issues are analyzed, therefore, further analyses would be interesting regarding this domain. A summary of the different studies regarding FLG knockdown in 3D models is available in Niehues et al. (102).

Interestingly, FLG silencing by mean of siRNAs in skin equivalents, cultivated for 14 days at the air–liquid interface, has been shown to render HSE more sensitive to the Th2 cytokines IL-4 and IL-13 (15 ng/ml of each when combined), increasing their effects on the model (93). These consequences include histological changes such as epidermal thickening and alterations in values of the superficial cutaneous pH measured. Furthermore, several effects induced by the deficiency in FLG expression, such as the upregulated expression of IVL or of occludin, were hampered by the addition of IL-4 and IL-13 (93).

Filaggrin-2 knockdown in RHE cultured for 11 days was also created by the use of shRNA production (103). Silencing FLG2 in keratinocytes of RHE led them to produce a thinner epidermis exhibiting parakeratosis, a compact cornified layer, and several alterations in the keratinocyte differentiation program including reduced processing of FLG, hornerin, corneodesmosin and reduced levels of caspase 14 and bleomycin hydrolase. A less acidic pH and an increased sensitivity to UVB was also attributed to FLG2 knockdown in RHE (103).

An alternative to the preparation of knockdown epidermal 3-D models has been developed by the use of keratinocytes that carry mutation(s) in genes of interest. Such methods avoid knockdown-derived off-target effects, but depend on availability of patient biopsies. For example, FLG-null keratinocytes can be obtained from ichthyosis vulgaris patients and be used to create pathological HSE that can be compared to healthy HSE (102). In this case, no alteration in the expression of keratin 10, IVL and transglutaminase-1 differentiation markers was reported, whereas some decrease in occludin and claudin-4 expression could be observed at protein level, affecting tight junctions. Despite such a decrease, no alteration in the epidermal barrier function could be reported using lucifer yellow and biotin permeation assays. Such FLG-null HSE were further analyzed after incubation with IL-4 and IL-13 (50 ng/ml for each cytokine), a treatment which again produces alterations in the expression of several differentiation markers (102).

Finally, explants from healthy and non-lesional human AD skin, harboring or not FLG mutations, have been cultured on dermal equivalents (104). Expression profile for most proteins remains identical in culture to their counterpart established *in vivo*, regardless of the presence of FLG mutations. This kind of model has allowed assessing that FLG mutations neither alter expression of the kallikrein 5 protease involved in cutaneous desquamation or the expression of the lympho-epithelial Kazal type related protease inhibitor (LEKTI).

### Skin Equivalents Using AD Cells

In order to understand potential cross talk between fibroblasts and keratinocytes in the context of AD, organotypic skin cultures consisting of fibroblasts derived from perilesional atopic skin and healthy keratinocytes, as well as healthy fibroblasts and atopic keratinocytes have generated for comparison with controls (105). That study demonstrated that atopic fibroblasts contribute to the particular pathological microenvironment of keratinocytes by releasing paracrine mediators which are responsible for hyperproliferation and reduced expression of differentiation markers in keratinocytes. Moreover, healthy fibroblasts have been shown able to rescue alterations in stratification and differentiation of keratinocytes as they are usually observed in AD epidermis. Interestingly, cocultures made of the two atopic cell types could not form any stratified epidermis in the model, the effects being partially dependent on expression of leukemia inhibitory factor by fibroblasts.

### Skin Equivalents Containing Lymphocytes

Many cytokines are being produced by lymphocytes. Thus, in order to better mimic the *in vivo* situation, as well as in order to create a model that is adequate to test drugs or other actives which could target together the epidermal and immune compartments, cocultures of keratinocytes and lymphocytes have been developed. Two different organotypic skin equivalents have been developed using HaCaT cells cocultured with lymphocytes (106). For both procedures, HSE were prepared using dermal matrix containing fibroblasts. In the first case, the epidermal tissue was removed from its dermal matrix and then cocultured in the presence of activated T cells. In the other procedure described by Engelhart and his colleagues (94), the whole HSE was transferred onto a collagen matrix containing the lymphocytes, coated with cell-free collagen mixture that acts as an adhesive for the HSE. Using this integrated organotypic skin model, spongiosis, keratinocyte apoptosis, reduced expression of E-cadherin, expression of intercellular adhesion molecule-1, upregulation of neurotrophin-4, as well as elevated levels of pro-inflammatory cytokines and chemokines were reported (94). In addition, this integrated epidermal model was used to show positive effects of anti-inflammatory drugs on the epidermal barrier through measurements of transepithelial electrical resistance.

Cross talk between T cells and keratinocytes has also been studied in a skin equivalent generated on de-epidermized dermis (107). In such model, CD4^+^ T cells were introduced between the transwell membrane and the dermal side of a fully developed skin equivalent. Th1 and Th17 polarization of the CD4^+^ T cells could be obtained and was able to induce psoriasis-like epidermal inflammation. However, induction of Th2 polarization of the CD4^+^ T cells was not successful, indicating that this model still requires refinements to adequately mimic features of AD.

## Conclusion and Perspectives

**Figure 2:**
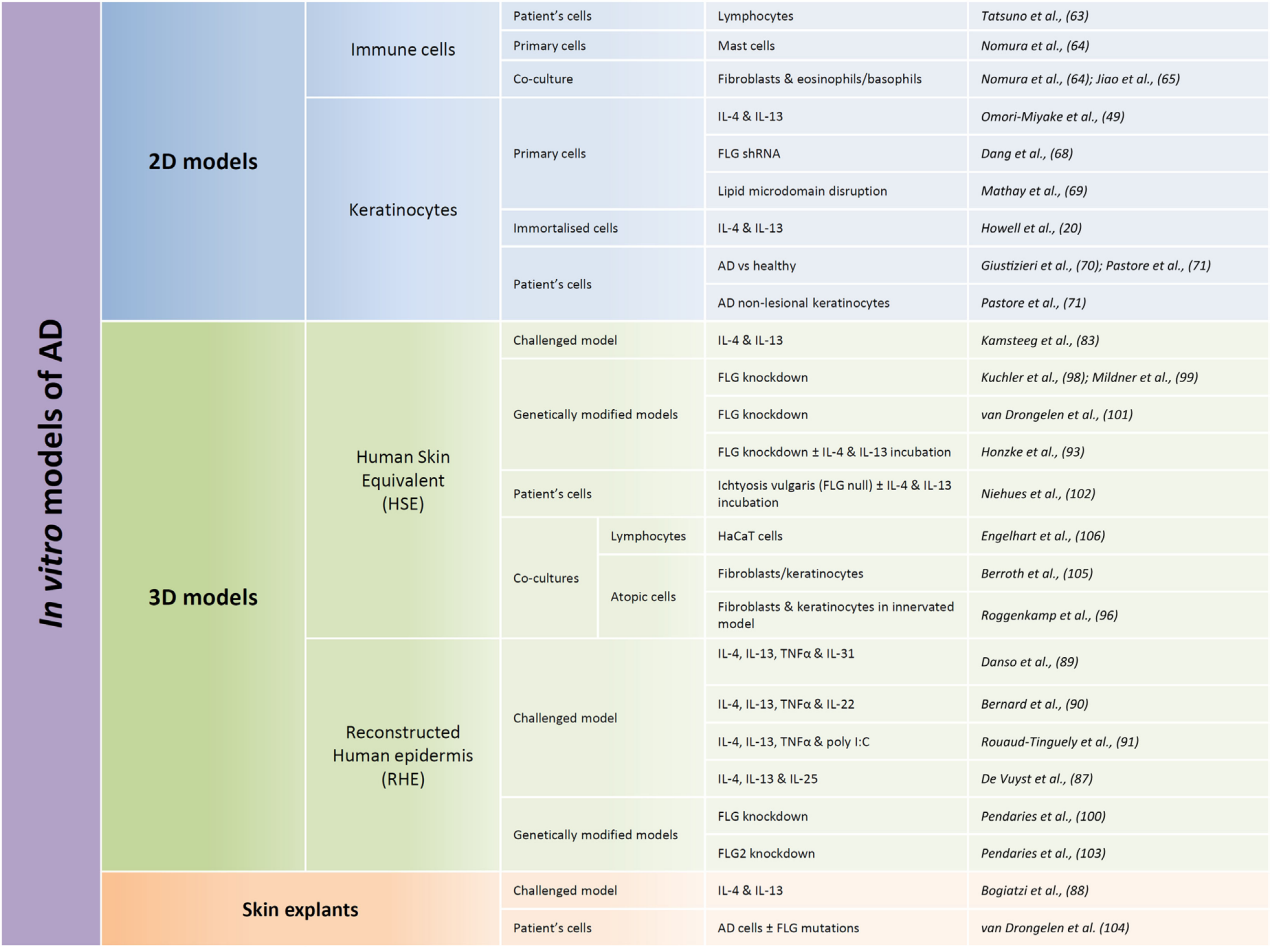
Summary of published models developed for *in vitro* studies of atopic dermatitis (AD) features. Different types of *in vitro* models can be used in regards to the research focus, from the most simple ones consisting of only one cellular type (named here 2D models) to more complex models reconstituting the different layers of the epidermis (called here 3D models). 2D models are used in order to characterize behavior of one given cellular type whereas 3D models are more commonly used with the aim of trying to reproduce some features of the pathology. Within these two types of models, immortalized cells can be used, but in order to be closer to the *in vivo* situation, primary cells are often used. Cells derived directly from patients are probably the most relevant but also the more complicated to obtain and the most limited. While performing 3D models, two different types of models are commonly used: human skin equivalents (HSE) or reconstructed human epidermis (RHE). HSE reproduce the epidermis on top of a dermis equivalent, whereas RHE only reproduce the different layers of the epidermis. HSE are more complicated to produce but display the advantage to present a dermal compartment whereas RHE allow evaluation of keratinocyte-specific type of responses and analysis of released or secreted molecules in the culture medium. HSE or RHE can both be challenged by interleukin cocktails and genetically modified. And cocultures with immune cells could be performed in both cases.

Several and quite various options for *in vitro* human models have been chosen to allow studies of AD pathogenesis (Figure 2). Every option uses different rationales aimed at creating alterations in otherwise normal tissues. They were also chosen for their potentiality in evaluating preventive and/or therapeutic treatments. To date, published studies highlight the requirement for therapeutic strategies which can target restoration of a functional epidermal barrier or which can inhibit T cells from overproducing cytokines. They also suggest that therapeutic approaches which could more specifically block the action of a particular cytokine, or of its receptor, are based on highly relevant data.

Interestingly, in aforementioned coculture models (106, 107), authors have shown that drugs acting on inflammation, like dexamethasone, tacrolimus, or cyclosporine A, are able to suppress inflammatory cell infiltrates by inhibiting the cytokine production triggered by T cells present in the model. Such drugs were also tested on HSE previously treated with IL-4 and IL-13; however, in this case, no improvement in epidermal spongiosis and no reduction in the expression of CA2 and NELL2 could be observed, very likely because of the absence of immune cells in the model (83). Thus, other treatments more specifically targeting epidermal features should now be tested to appreciate the interest of immune-free models.

It is, however, important to keep in mind that these *in vitro* models represent a simplification of the *in vivo* situation as they remain models only and that it will probably never be possible to reproduce all human features of the pathology in such models. Furthermore, it is also worth to mention that different features observed in AD skin, at the histological level as well as at the molecular level, could refer to general inflammation or epidermal hyperplasia. It is, therefore, required to combine a panel of various readouts but in general, finding the “best” readouts in a biological or pathological model is never an easy task. Therefore, regarding AD, in order to help scientists to set up correct and efficient readouts, several studies have used keratinocytes derived from AD patients and compared them with cells from healthy, or even psoriatic, keratinocytes in order to identify specific transcriptomic or proteomic patterns (108–113). Another important issue to take into account about readouts is the type of model that is used. Indeed, cross talks between the different cell types of the model, like keratinocytes and fibroblasts, may influence significantly the results. For example, fibroblasts in the dermal matrix can improve keratinocyte differentiation while sustaining epidermal viability (114). In addition, fibroblasts can also produce TSLP (35). Thus, when fibroblasts are included in the dermal matrix of HSE, a special attention must be paid to take this factor into account when assessing hypothesis and drawing conclusions. Furthermore, environmental stress conditions are known to play a role in the development of AD [for extensive review see Ref. (8)]. Intensive use of soap (4), proteases from house dust mites, exposure to pollutants and chemicals (4, 115), and psychological stress (116, 117) notably have been reported as risk factors able to play some role in the development of AD. It could be envisaged to add topically soap, detergents or house dust mites to *in vitro* models in order to mimic some environmental factors that could play a role in the development of AD. However, factors like psychological stress are less obvious to integrate into an *in vitro* model, also because underlying mechanisms are still incompletely understood.

Despite *in vitro* studies are performed on simplified models, they might nevertheless bring better understanding of a pathology, while trying to answer rather basic questions. As an example, a typical morphological feature of AD inside the epidermis is the appearance of intercellular spongiosis. Spongiosis is a well-known characteristic of this disease but is not yet fully understood. It is characterized by loss of cohesion between keratinocytes together with some influx apparently associated with an accumulation of hyaluronan (HA) (118, 119). HA is indeed reported as being increased in intercellular spaces of lesional AD epidermis *in vivo*, in simultaneity with an increased expression of the HA synthase HAS3 (119, 120). Interestingly, enhancement of HA synthesis and deposition in RHE is also simultaneous with treatment of the epidermis with Th2 cytokines, especially with IL-4 treatment performed after cholesterol depletion from keratinocyte plasma membrane (87, 121). Whether any relationship exists between epidermal regulations of HA concentration (120, 122) and spongiosis observed during AD would be interesting to investigate. Is the elevated HA concentration a cause, or a consequence of spongiosis? This question could be answered using *in vitro* models where HA could be withdrawn by mean of incubation with a hyaluronidase that degrades HA into small fragments, or by inhibiting epidermal HA synthesis, either pharmacologically or by means of siRNA or shRNA targeting the expression of HAS enzymes.

It would be important to further investigate implication of Th17 and Th22 cells in the context of AD as the classical paradigm of AD as a “Th2” disease has recently been challenged. Indeed, Th17 is mostly associated with psoriasis as it is more induced in psoriasis than in AD (123). However, Th17 cells have been demonstrated in AD and associated with severity of the disease (124, 125). IL-17 expression was found at higher levels in acute AD skin lesions than in chronic lesions or healthy skin, even if in a lesser extent compared with psoriasis lesions (123). Interestingly, IL-17 could also negatively regulate expression of FLG as well as of tight junction proteins ZO-1 and ZO-2, desmosomes, e-cadherin, and various integrins (25). IL-17 is a key inducer of expression of antimicrobial genes (123), potentially explaining why psoriasis patients are less frequently subjected to skin infections than AD patients. Regarding Th22 cells, these are also reported as increased in AD skin and correlated with disease severity (126). IL-22 would, in RHE, affect expression of FLG, induce epidermal hyperplasia, and upregulate expression of pro-inflammatory molecules belonging to the S100 family of calcium-binding proteins (127). Further interesting, IL-17 and IL-22 could act synergistically to regulate notably gene expression of AMPs (128) and *S. aureus*, that frequently affects AD patients, could induce production of Th17 cells (129). In summary, the understanding of the pathogenic mechanisms is still in progress and further research concerning this area is needed. Further, more data regarding comprehension of the transit from the acute to the chronic stage of the disease and *vice versa* would also be useful.

To conclude, several skin models can mimic *in vitro* the pathological situation of AD, especially at particular points of view. However, no model is currently able to entirely resume *in vitro* the overall characteristics of AD. Therefore, investigations on this disease have to take into account the balance between advantages and disadvantages of the multiple available models before choosing the most appropriate one(s) and before collecting data in relation with specifically targeted scientific issues.

## Author Contributions

EV, MS, CE, CR, and YP shared comments to construct the manuscript that was drafted by EV. All authors have read and approved the final version.

## Conflict of Interest Statement

The authors declare that the research was conducted in the absence of any commercial or financial relationships that could be construed as a potential conflict of interest.

## Abbreviations

AD, atopic dermatitis; IL, interleukin; TSLP(R), thymic stromal lymphopoietin (receptor); LOR, loricrin; IVL, involucrin; RHE, reconstructed human epidermis; HSE, human skin equivalent; ECM, extracellular matrix.
